# Negative pressure therapy stimulates healing of critical-size calvarial defects in rabbits

**DOI:** 10.1038/bonekey.2013.33

**Published:** 2013-04-03

**Authors:** Larry D Swain, Douglas A Cornet, Michael E Manwaring, Barbara Collins, Vinay K Singh, Dan Beniker, David L Carnes

**Affiliations:** 1 Kinetic Concepts Inc., San Antonio, TX, USA; 2 Biomedical Engineering, The University of Texas at San Antonio, San Antonio, TX, USA; 3 Biomedical Engineering, The University of Texas Health Science Center at San Antonio, San Antonio, TX, USA; 4 Department of Periodontics, The University of Texas Health Science Center at San Antonio, San Antonio, TX, USA

## Abstract

Negative pressure therapy (NPT) is the controlled application of subatmospheric pressure to wounds. It has been shown to stimulate healing across a broad spectrum of soft-tissue wounds, at least in part from the application of mechanical stress on cells and tissues in the wound environment. This study tests the hypothesis that application of NPT to cranial critical-size defects (CSD) in skeletally mature rabbits leads to osseous healing. NPT was delivered 1, 4, 6 or 10 days over CSD-containing calcium phosphate scaffolds placed in contact with intact dura. At 12 weeks after defect creation, NPT groups exhibited significantly greater defect bridging and bone within the scaffolds (*P*<0.01). Increasing duration of NPT did not result in a greater amount of bone within the scaffolds, but did increase the amount of bone distributed in the upper half of the scaffolds. Appearance of tissue within defects immediately following the removal of NPT at day 6 suggests alternating regions of dural compression and distention indicative of cell stretching. Dura and adjacent tissue were composed of multiple cell layers that extended up into the scaffolds, lining struts and populating pore spaces. An extracellular matrix densely populated with cells and capillaries, as well as larger vessels, infiltrated pores of NPT-treated scaffolds, while scattered spindle-shaped cells and sparse stroma are present within pores of control scaffolds. This rabbit model data suggest that NPT activates within mature dura a natural healing cascade that results in osseous tissue formation without the addition of exogenous factors or progenitor cells.

## Introduction

Congenital anomalies, trauma and diseases require significant numbers of craniofacial surgical interventions each year. Unfortunately, older children and adults demonstrate markedly attenuated repair mechanisms that essentially restrict calvarial repair to infants.^1^ Research using a critical-size defect (CSD) model, so named because the defects do not heal, clearly demonstrates the limited regenerative capacity of skeletally mature cranial membranous bone.^2^,^3^ Successful treatment can be achieved using autologous bone marrow grafting, but the procedure is limited by the amount of graft material available for harvest, as well as donor site morbidity and pain. The requirement for autologous bone marrow grafts or other osteoinductive approaches suggests that the cells and factors required for healing are either absent from the injured site or do not respond to trauma or surgical intervention in such a way as to initiate healing.

Research indicates that progenitor cells necessary for osteogenesis reside in the dura underlying the defect, and, in adult animals and humans >2 years of age, trauma does not stimulate the proliferation and differentiation of these progenitors.^4^,^5^ Nevertheless, these cells have not lost their capacity to differentiate. Indeed, treatment with allogenic demineralized bone matrix stimulates healing of CSD in mature rats and non-human primates, and the source of the osteogenesis is the progenitor cell population from the dura.^6^,^7^,^8^,^9^ Unfortunately, the use of demineralized bone matrix is associated with wide variation in healing outcome, as well as negative host response and morbidity.^10^ Ideally, what is required for calvarial healing is a reliable method for predictably stimulating, *in situ*, progenitor cells in the dura to initiate the osteogenic healing pathway without the requirement of exogenous cells or factors.

Skeletal tissues respond to a variety of mechanical stimuli that regulate the proliferation and differentiation of mesenchymal stem cells and committed osteogenic precursors, as well as osteoblasts and osteocytes themselves. Recent publications suggest that altering the physical environment of skeletal tissue by mechanical means promotes healing.^11^ These reports led us to hypothesize that the application of negative pressure may effectively stimulate osseous healing in the CSD model. Negative pressure therapy (NPT) is the controlled application of subatmospheric pressure to a wound site delivered via a wound dressing that acts as a pressure manifold.^12^,^13^ NPT stimulates healing across a broad spectrum of soft-tissue wounds, at least in part from the application of mechanical stress on cells and tissues in the wound environment.^14^ The application of stress results in stretching at the tissue and cell level that stimulates cell proliferation and differentiation required for healing via modulation of the extracellular matrix by cell-mediated mechanical signaling mechanisms.^15^

The immature dura is the source of the osteoprogenitor cells that form the calvaria during embryonic development and perinatal growth. It is likely that mechanical stress on the dura from the growing brain stimulates the proliferation and differentiation of these osteoprogenitor cells.^5^,^16^,^17^ Therefore, we reasoned that the application of negative pressure to CSD may result in the transmission of forces to the underlying dura, with concomitant cell stretching, stimulating osseous healing in a manner analogous to the osteogenic response in immature dura induced by the growing brain. The experimental data herein reported is the first to examine the effects of NPT in bony tissues. We therefore chose to use the pressure established as most effective for soft-tissue wounds, −125 mm Hg.^18^,^19^ Thus, this study was designed to test the hypothesis that application of NPT to cranial CSD in skeletally mature animals leads to osseous healing.

## Results

### Defect healing at 12 weeks

**Figure 1:**
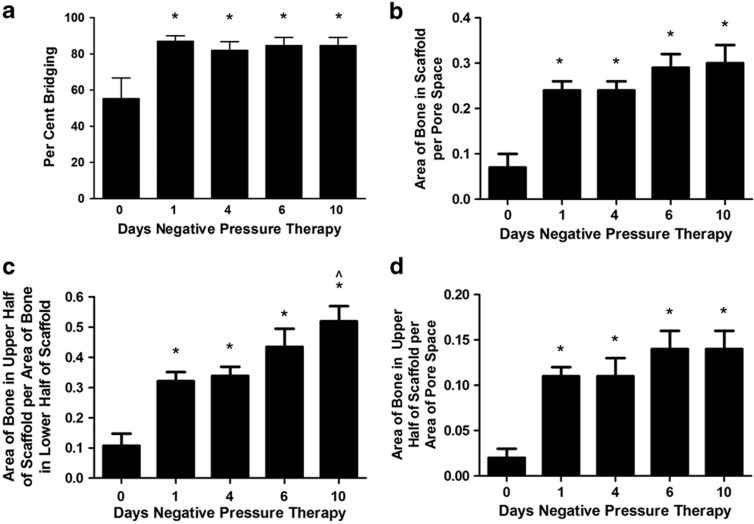
Image analysis of histology. (**a**) New bone bridging the CSD: the amount of new bone bridging the CSD expressed as a percentage of the diameter of the defect was significantly greater in the NPT groups compared with the 0 treatment group. (**b**) New bone as a function of scaffold pore space: there was significantly more new bone within the pore space of scaffolds in the NPT groups compared with new bone within the pore space of scaffolds in the control group. Data are expressed as the area of total new bone per area of total pore space. (**c**) Ratio of new bone in upper half of scaffold to new bone in the lower half of scaffold: the area of new bone in the upper half of the scaffold expressed as a function of the pore space in the upper half of the scaffold was divided by the area of new bone in the lower half of the scaffold expressed as a function of the pore space in the lower half of the scaffold. (**d**) New bone as a function of scaffold pore space in the upper half of the scaffold: There was significantly more new bone within the pore space of the upper half scaffolds in the NPT groups compared with new bone within the pore space of the upper half of the scaffolds in the control group. Mean±s.e.m.. *Different from 0 treatment group at *P*<0.01; ^Different from 1- and 4-day treatment groups at *P*<0.05.

Histomorphometry of new bone within the calvarial CSD indicates that NPT had a positive effect on healing. At 12 weeks, bridging of the defects with new bone was significantly greater (*P*<0.01) in the NPT groups than in controls (Figure 1a).

Similarly, at 12 weeks, all NPT groups exhibited significantly more bone within the scaffold than did the control group (*P*<0.01). The mean area (±s.e.m.) of pore space within the scaffolds in all defects was 31.86±0.43 mm^2^. The mean area of new bone within this scaffold pore space in the control defects was 3.76±0.87 mm^2^, while the area of new bone in the NPT groups ranged from 6.90±0.74 to 7.64±0.66 mm^2^ (1.8- to 2.0-fold increase). These data are displayed as a function of available pore space in Figure 1b. Interestingly, increasing the duration of NPT, even by 10-fold, did not result in a significantly greater amount of new bone within the scaffolds of these defects, suggesting that the major inductive effects of NPT were initiated within the first 24 h of application.

**Figure 2:**
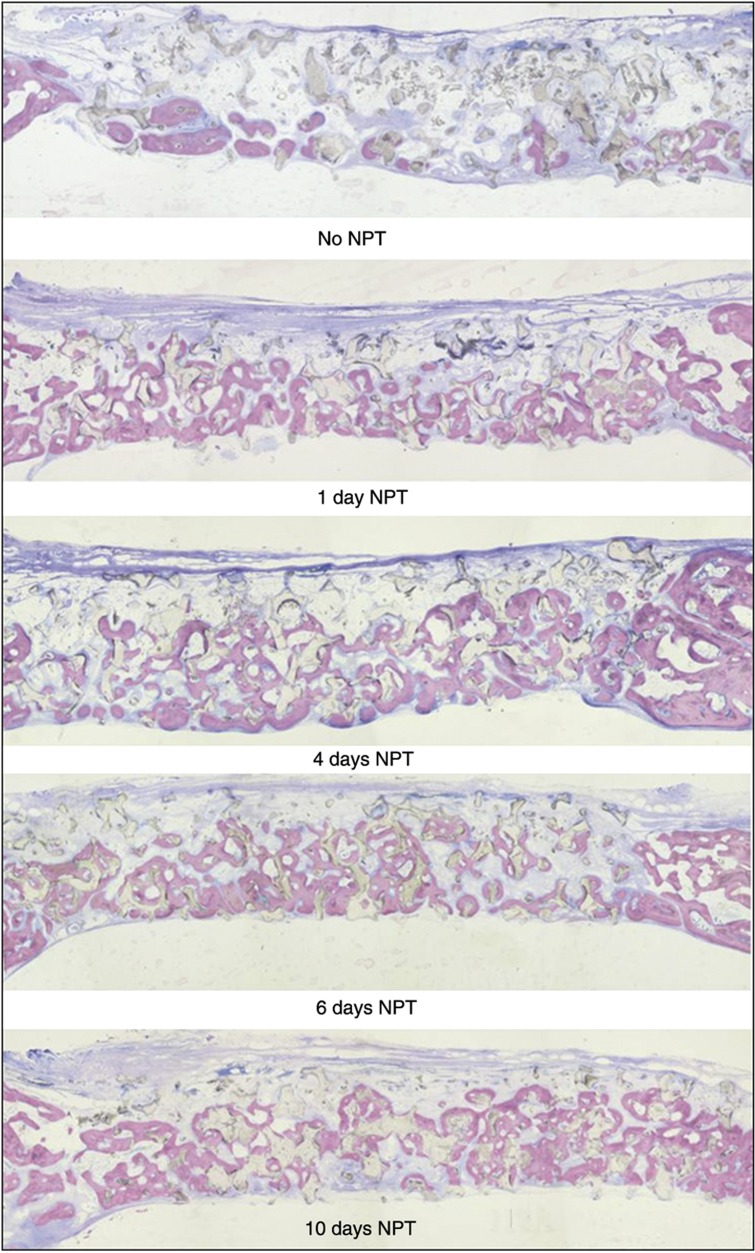
Micrographs of new bone within the scaffolds. Representative undecalcified plastic-embedded sections stained with Sanderson's rapid bone stain. Top to Bottom: 0 (No NPT), 1, 4, 6 and 10 days NPT. The specimens were harvested 12 weeks after creation of the CSD. Osteoid cells within the marrow and soft tissues are stained blue. Mineralized bone is stained pink. Residual scaffold is light brown. Original magnification × 5.3.

Visual examination of micrographs suggests that not only was more new bone present in the scaffolds from NPT groups compared with the control group but also that this new bone extended throughout the thickness of the scaffolds (Figure 2).

Analysis of the new bone in the NPT defects revealed that while there was no correlation between the total area of new bone within the scaffolds and the duration of therapy, there was a marked difference in the amount of the new bone within the upper half of the scaffold. Specifically, a linear relationship (*R*^2^=0.93, slope significantly different from 0 at *P*<0.36) exists between the duration of NPT and the ratio of new bone in the upper half of the scaffold compared with new bone in the lower half in contact with the dura (Figure 1c). The actual amount of new bone distributed in the upper half of the scaffolds ranges from 4.8 times that of control after one day of NPT to 6.5 times the control amount after 10 days of treatment (Figure 1d).

These data suggest that while one day of NPT was sufficient to initiate significant new bone induction and bridging, increasing the duration of NPT altered the distribution of bone within the scaffold, thereby overcoming the limitations of tissue distribution within a scaffold attributable to passive diffusion mechanisms.

### Early effects at the scaffold–dura interface immediately following 6-day NPT

**Figure 3:**
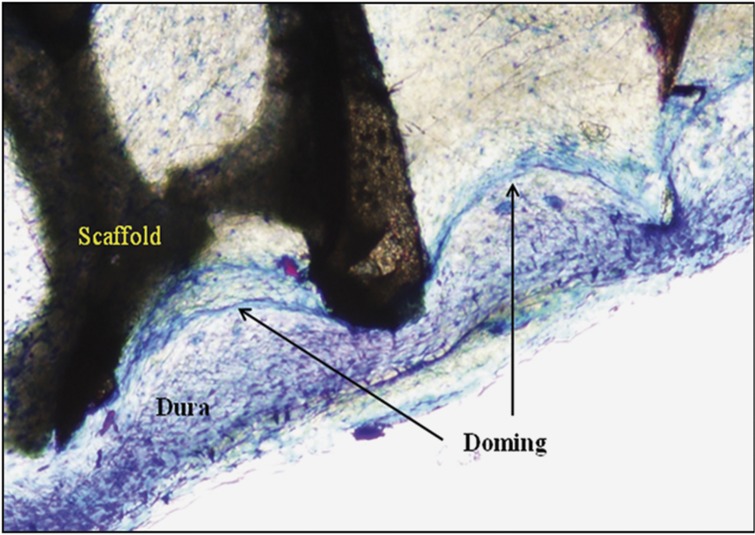
Micrograph of tissue doming at the scaffold–dura interface immediately after 6 days of NPT. Undecalcified plastic-embedded section harvested immediately following 6 days of NPT and stained with Sanderson's rapid bone stain. Original magnification × 10.

Histologic examination of specimens prepared from the subset of animals that received NPT for 6 days, and then were immediately euthanized, revealed undulations of the dura coincident with the scaffold–strut interface. As displayed in Figure 3, the tissue at the dura–scaffold interface resembles a series of domes, each extending into the space between adjacent scaffold struts. The appearance of the tissue is consistent with the transfer of pressure/tensile force to the dura through the scaffold, resulting from the application of negative pressure delivered through the scaffold. The morphology suggests alternating regions of dural compression and distention indicative of stretching at the cellular level.

**Figure 4:**
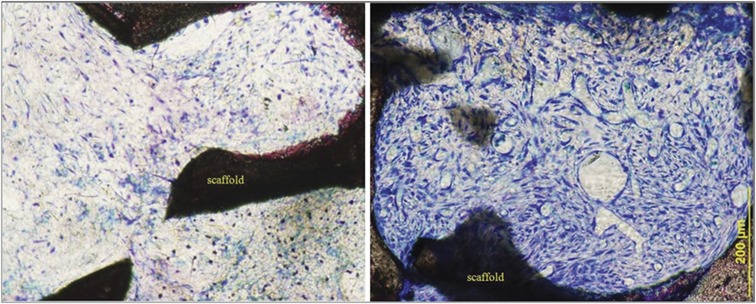
Micrographs comparing cell density and vascularity within the scaffold pore space. Undecalcified plastic-embedded sections stained with Sanderson's rapid bone stain. The specimens were harvested immediately after 6 days of NPT to the CSD. A fibrous connective tissue with scattered spindle-shaped cells and sparse stroma is present within the pores of the control scaffold (left panel). A mature extracellular matrix densely populated with cells and an abundance of capillaries, as well as larger vessels, has infiltrated the pores of the NPT-treated scaffold. Original magnification × 10.

**Figure 5:**
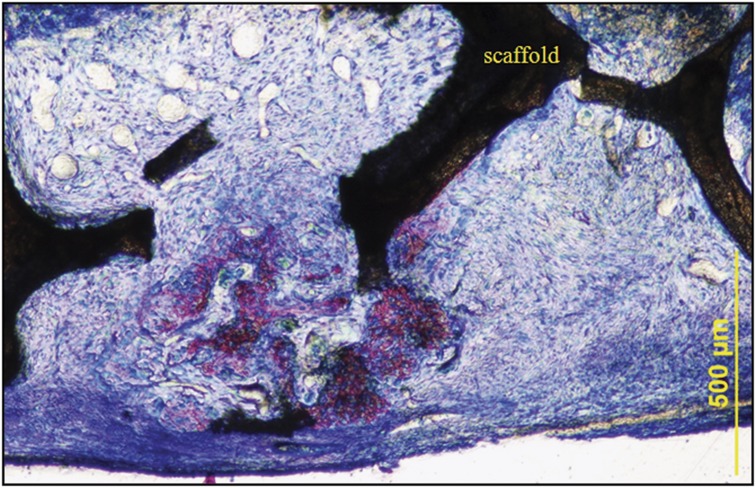
Micrograph of the dura–scaffold interface. Undecalcified plastic-embedded section stained with Sanderson's rapid bone stain. The specimens were harvested immediately after 6 days of NPT to the CSD. Note the dense, multilayer morphology of the dura directly beneath the scaffold. Bone formation is present even at this early time point. Original magnification × 10.

Greatly increased vascularity immediately after NPT compared with the 0 treatment group is also apparent (Figure 4). The pore space is populated not only with capillaries and microvessels but also with an abundance of larger vessels, suggesting that, in addition to angiogenesis, vascular invasion of the scaffold by pre-existing vessels has occurred. It is also apparent that there is a marked difference between the appearance of the cells and extracellular matrix populating the interstitial spaces throughout the thickness of scaffold in the NPT group compared with the untreated control group. A fibrous connective tissue with scattered spindle-shaped cells and sparse stroma was present within the pores of the control scaffold, while a mature extracellular matrix densely populated with cells and an abundance of capillaries, as well as larger vessels, had infiltrated the pores of the NPT-treated scaffold. In NPT-treated animals, the dura and immediately adjacent tissue were increased in thickness, being composed of multiple cell layers that extended up into the scaffold, lining the surface of the struts and populating the pore spaces (Figure 5). The presence of new bone even at this early 6-day time point suggests the migration of mesenchymal cells from the dura that differentiate to functional osteoblasts and osteocytes.

## Discussion

Our data are consistent with the concept that negative pressure activates within the mature dura, a pattern of osseous tissue formation normally active only during embryonic development and growth of the calvaria. Interestingly, this activation was accomplished without a requirement for the addition of exogenous factors, such as demineralized bone matrix, its active component, bone morphogenetic protein or a population of progenitor cells usually supplied by autologous grafting. CSD created in skeletally mature rabbits treated with negative pressure in this study demonstrate osseous healing, which suggests differentiation of progenitor cells directly into the bone. The literature supports this concept that progenitor cells from the dura differentiate directly to osteoblasts that synthesize new bone matrix with subsequent mineralization.^20^ Cells derived from bone surrounding the defect do not contribute significantly to the healing process.^21^,^22^ Multiple mechanical stimuli in spatiotemporal concert are known to regulate proliferation and differentiation of mesenchymal progenitor cells.^11^,^23^ Our study suggests that physical alterations in the extracellular environment induced by NPT may modulate these biomechanical stimuli. The relevant question, then, is what are the mechanisms by which negative pressure initiates the osteogenic cascade in the absence of added exogenous factors and/or cells?

The observation that the ability of the calvaria to heal or regenerate is limited to juvenile animals led to studies that determined immature dura supported osseous healing, while dura from adult animals did not.^24^,^25^ Stretching of the dura as a consequence of the rapid increase in the size of the brain during growth produces stretching at the cellular level that activates proliferation and differentiation of progenitor cells in the immature dura.^5^,^16^,^17^ Tensile forces on the dura are significantly reduced in concert with slowing of brain growth, resulting in a markedly attenuated stimulus for osteoprogenitor cell proliferation and differentiation in mature dura.^16^ Experiments have demonstrated that stretching dural cells does indeed activate these processes and, in the case of osteoprogenitors, results in the elaboration of factors and matrix molecules that are diagnostic for osteoblast differentiation.^16^ Tensile strain induces osteoblasts not only to proliferate but also to increase osteogenic growth factor production, extracellular matrix deposition and assume an ‘angiogenic' role via enhanced vascular endothelial growth factor production.^5^ Application of NPT increases the expression of vascular endothelial growth factor and fibroblast growth factor-2, as well as increased local release of interelukin-6 and -8.^19^,^26^,^27^,^28^ The results of this study suggest that negative pressure application may likewise mediate dural stretching with subsequent strain-induced activation of osteoprogenitor cells and their subsequent differentiation.

Application of mechanical forces to soft-tissue wounds results in tissue deformation that leads to cell stretching. Cells respond to such stretching by regulating specific genes and/or the induction of cellular pathways that lead to tissue-specific cell differentiation and angiogenesis.^29^,^30^ Following application of negative pressure to complex soft-tissue wounds, tissue morphology at the therapy–tissue interface is characterized by doming and has been described by Saxena *et al*.^15^ as ‘an undulating contour of protrusions and indentations corresponding to the geometry of the NPT OCF [open-cell foam] in contact with the wound tissue'. Finite-element analysis of wounds undergoing NPT reveals that forces transmitted to the tissue are within the range of physiologically relevant values known to stimulate cell proliferation and differentiation.^15^,^31^ Strikingly, the morphological profile of the dura at the scaffold–dura interface in this study is remarkably similar to the appearance of soft-tissue wounds undergoing NPT. There is an apparent doming of tissue into the spaces between the scaffold struts that may be entirely compatible with the transduction of force to the dura sufficient to stimulate the proliferation and differentiation of osteoprogenitor cells. External application of such forces has been shown to stimulate cell proliferation.^32^

Nevertheless, it is apparent from this study that factors in addition to tissue stretching may be involved in the osseous healing response and that the benefits of mechanostimulation are not restricted to deformation of soft tissues. This is evident from the appearance of the dura at the scaffold–tissue interface in control defects that were not stimulated with negative pressure. The undulating contour of protrusions and indentations in the dura underlying these defects suggests that closure of the skin may also result in the transmission of compressive forces through the scaffold that induce tissue stretching. In addition, calcium phosphate surfaces have been shown to promote osteogenic differentiation of bone marrow-derived mesenchymal stem cells.^33^,^34^,^35^ Irrespective of these observations, osseous healing in control defects is significantly less compared with NPT-treated defects, suggesting that additional mechanisms attributable to the application of negative pressure are initiated, consistent with known mechanisms of action of NPT. Scherer *et al*.^36^ have shown that tissue strains induced by a mechanically compressed reticulated open-cell foam (ROCF) manifold at a wound surface, in the absence of negative-pressure-induced fluid flow, are insufficient to stimulate mechanically cell proliferation. Accordingly, a factor contributing to increased osseous healing in the NPT-treated defects may be mechanically induced fluid flow. That such flow occurs at the wound site is verified by the collection of fluid in a small canister that is a component of the unit creating the vacuum. It is well established that application of NPT results in increased fluid flow through the interstitial spaces of soft-tissue wounds.^13^,^36^ Interstitial fluid flow is the primary mechanism by which bone cells perceive changes in their mechanical environment, and strain-induced fluid flow regulates bone cell anabolic response to mechanical cues.^23^,^37^,^38^ Riddle *et al*.^39^ have recently shown that mesenchymal stem cells are stimulated to proliferate and differentiate along osteoblastic pathways via fluid-flow-induced mechanisms, suggesting regulation of both osteoblast and mesenchymal precursors via a common signaling pathway. NPT-induced fluid flow in treated defects may contribute to stimulation of osteoprogenitor cells present in dura, thereby producing the enhanced osseous healing observed in this study.

Recent studies have shown that discrete periods of mechanostimulation activate tissue-specific progenitor cell pathways that continue to influence skeletal tissues beyond the period of mechanostimulation.^11^ These discrete periods can range from <1 h to several days, suggesting wide variation in the required duration of a physical stimulus sufficient to achieve a threshold between quiescence and activation. Threshold behavior is recognized in bone, whereby minimal cell and tissue responses are observed until a threshold is achieved, after which incremental increases in stimulus elicit nonlinear increases in response.^40^ Our data indicate that exposure to negative pressure for as little as 24 h significantly enhanced osseous healing compared with untreated controls. It is important to note that regardless of the length of time the defects were exposed to negative pressure, there was virtually complete bony bridging of the defect on the calvarial surface of the dura (Figure 2). Nevertheless, longer exposure to negative pressure (up to 10 days) resulted in osseous healing characterized by a more uniform distribution of bone throughout the scaffold thickness, demonstrating that, as suggested by Morgan *et al*.,^11^ the physical environment can be manipulated to promote a specific healing response.

Induced fluid flow may contribute to this more uniform distribution of bony tissue. A fibrin-like matrix is observed throughout the NPT-treated scaffolds, independent of therapy time. Research has shown that mechanical connections transmit cues that are necessary for cells to respond to chemical signals.^29^ It is the extracellular matrix that forms the mechanically continuous structure with which the cytoskeleton of cells connect through cell surface structures, such as integrins, which are required for proliferation, differentiation and migration.^30^ The uniform distribution of a fibrin-like network that extends throughout negative pressure-treated scaffolds, presumably as a result of induced, directional fluid flow, may contribute to the extracellular matrix necessary to support the migration of progenitors up into the scaffold and their subsequent differentiation. Directional fluid flow induced by NPT may establish gradients in extracellular matrix density and protein distribution that are prolonged over the course of treatment. Tissue dilatation, altering extracellular matrix densities, has been identified as a mechanical stimulus for tissue differentiation.^11^ Tissues distending into the pores of the ROCF manifold in NPT-treated soft-tissue wounds, or, in this study, dural tissue distending into pores of the scaffold, represent regions that experience triaxial tensile-strain-induced dilatation.^29^

Mammoto *et al*.^41^ have recently reported a transcriptional mechanism sensitive to both mechanical and chemical cues that controls angiogenesis, strengthening the link between mechanical force and capillary growth.^42^ Clinical application of NPT has been shown to stimulate capillary formation, with subsequent wound healing, in diabetic, debilitated patients.^43^ In this study, the initial healing response induced by NPT is, likewise, characterized by the presence of many capillaries and small blood vessels. Interestingly, other mechanisms exist for providing functional circulation independent of endothelial sprouting or proliferation. Tissue tension can induce and direct rapid migration of functional, pre-existing vasculature.^44^ This process apparently requires extracellular matrix deformation as a stimulus for vessel translocation. Application of NPT may support this phenomenon by providing the biomechanical force necessary to mediate vessel translocation. Indeed, Kilarski *et al*.^44^ suggest that biomechanically driven vascular translocation may contribute to the enhanced granulation tissue formation and accelerated wound healing associated with NPT.

A growing body of evidence demonstrates a close temporal and spatial relationship between angiogenesis and osteogenesis. Schipani *et al*.^45^ have reviewed this relationship and concluded that increased vascularization leads to increased delivery of skeletal stem cells and elevated levels of endothelial cell-derived osteogenic growth factors. In addition, rapid population of scaffolds with pre-existing, functional vessels independent of the angiogenic process, as well as mechanosensitive/responsive angiogenic pathways, may converge to facilitate greatly nutrient/waste exchange, thereby overcoming the well-known difficulties of integrating scaffolds into host bone related to the limitations of passive diffusion.

In summary, application of NPT resulted in sustained osseous healing in the critical-size rabbit calvarial wound model. Our data suggest that negative-pressure-induced mechanical signals (tissue stretching) and fluid flow, as well as angiogenesis and vessel translocation, combine to stimulate new bone formation within the defects, acting to trigger, *in situ*, a natural healing cascade without requirement for exogenous cells and/or growth factors. Our data are consistent with the concept that NPT over CSD created in skeletally mature rabbits activates a pattern of osseous tissue formation normally only active during embryonic development. This pattern is characterized by differentiation of progenitor cells directly into bone. Thus, multiple mechanical stimuli may function in spatiotemporal concert to initiate osteogenesis and vascularization that promote sustained osseous healing, and suggest the possibility of tailoring negative pressure and temporal parameters to tissue-specific wound site requirements.

## Materials and methods

### Surgical procedure

**Figure 6:**
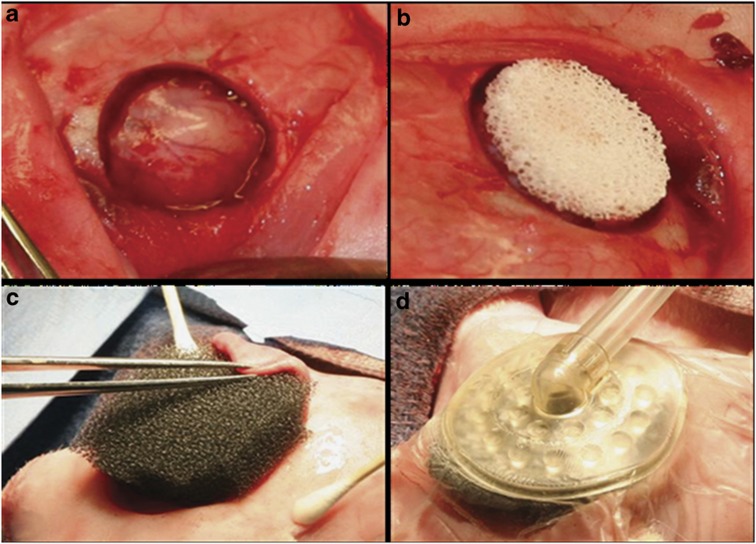
Surgical procedure. (**a**) Defect with intact dura, (**b**) defect with scaffold in place and (**c**) defect with ROCF dressing in place on top of scaffold as shown in (**b** and **d)**. (**d**) Defect with pad and tubing in place over ROCF.

**Figure 7:**
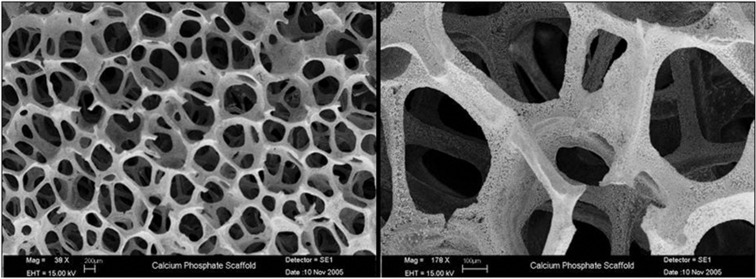
Scanning electron micrograph of scaffold.

Following Institutional Animal Care and Use Committee review, male, skeletally mature New Zealand White rabbits were clipped and prepped for aseptic surgery. A midline skin flap was raised over the parietal bones and reflected caudally, exposing the midsaggital and transverse sutures. Periosteum was incised along the midsaggital suture and the right or left transverse suture (randomized), and removed to expose the parietal bone. A 15 mm diameter CSD^2^ was created using a trephine at 13 000 r.p.m. under copious saline irrigation. A bi-cortical section of bone was removed to expose the dura mater, with great care taken to maintain dural integrity (Figure 6a). A 15 mm diameter, 3 mm thick custom-made high-porosity calcium phosphate scaffold that exhibits microhydroxyapatite surface characteristics^33^ was placed in contact with intact dura in both control and NP-treated wounds (Figure 6b). These scaffolds contain fully interconnected pores with a void volume >90% (Figure 7).

Inclusion into a control or experimental group was determined by a randomization protocol. In control animals, the skin flap exposing the parietal bone was immediately repositioned over the scaffold-containing defect and secured with surgical staples. The animals were recovered and returned to their cages. For experimental animals, Telfa Clear non-adherent, porous film (Covidien, Mansfield, MA, USA) was placed over the wound and scaffold. A 1 cm thick layer of polyurethane-reticulated, open-cell foam (ROCF; V.A.C. GranuFoam Dressing; KCI USA Inc., San Antonio, TX, USA) was positioned over the defect site (Figure 6c). The underside of the skin flap previously reflected to expose the parietal bone was placed in contact with the distal margin of the ROCF dressing. The ROCF dressing serves as a manifold to evenly distribute the applied negative pressure to the wound bed and scaffold.^15^,^36^ The ROCF dressing was then covered with a semiocclusive, adhesive polyurethane film (V.A.C. Drape, KCI USA Inc.) confining the application of negative pressure to the wound/scaffold site. Surrounding skin was prepped with Hollister medical grade adhesive (Hollister Inc. Libertyville, IL, USA) before drape placement for added adhesion. A 5 mm hole was made in the drape, centric with the scaffold, to allow communication with the negative pressure source. An adhesive T.R.A.C. Pad (KCI USA Inc.) with tubing was placed over the hole (Figure 6d) and then connected to a V.A.C. Freedom Therapy Unit (KCI USA Inc.) set to maintain a wound pressure of negative 125 mm Hg gauge pressure. Any leaks were sealed with additional pieces of V.A.C. Drape.

Animals were then tethered to the cage and pump via Lomir small animal jackets (Lomir Biomedical Inc., Malone, NY, USA) fitted with stainless-steel flex tubes and proprietary multilumen swivels. This afforded the animals full mobility within the cage without interrupting the delivery of negative pressure. Wound effluent was collected in a canister within the pump system.

NPT was delivered for 1, 4, 6 or 10 days in experimental animals per the randomization protocol (*n*=8 per group). At the conclusion of therapy, the animal was sedated and all NPT dressings except the scaffold were removed. The skin flap previously reflected to expose the parietal bone and maintained in contact with the distal margin of the ROCF dressing during NPT delivery was repositioned over the scaffold-containing defect and secured with surgical staples. Of note was the fact that the flap remained viable throughout therapy delivery and only minor edge freshening was required before closure. A subset of control and 6-day therapy animals were euthanized immediately following removal of NPT at day 6. All other control and experimental animals were maintained with food and water *ad libitum* until euthanized 12 weeks from the day of defect creation.

After euthanizing, the parietal bones were harvested and placed in 10% neutral-buffered formalin. Fixed calvaria sections were processed for plastic embedding. Specimens were dehydrated through a graded series of ethanols, defatted in acetone and returned to 100% ethanol. Samples were then infiltrated with increasing concentrations of Technovit 7200 (Exakt Technologies, Oklahoma City, OK, USA) in ethanol. Benzoyl peroxide (Sigma-Aldrich Co., LLC, St Louis, MO, USA) was added to the final infiltration step at a concentration of 1% and samples placed into appropriate-sized molds for polymerization via an Exakt 520 Light Polymerization Unit (Exakt Technologies). Following polymerization, individual specimens were bisected along randomly chosen diameters using an Exakt 300CP Band System (Exakt Technologies). A bisected half was chosen at random and a 3 mm slice cut. This slice was mounted such that the cut surface running through the defect center was against a histologic slide. From this, a 300 μm section was produced using the Exakt band saw. This section was then transferred to an Exakt 400CS Grinding System (Exakt Technologies) and ground to ∼30 μm thickness. Ground and polished sections were then stained via Sanderson's rapid bone stain (Surgipath, Richmond, IL, USA) with acid fuchsin counterstain.^46^

### Histomorphometric analysis

Histology slides of explanted defects in cross-section were photographed. Photographs were sequential and covered the entire defect region. Individual images were then imported into Adobe Photoshop CS2 (Adobe, San Jose, CA, USA) and merged to give a composite image. From this composite image, bone and scaffold were isolated and reduced to a bicolor image highlighting new bone growth and residual scaffold material. The resulting bicolor image was exported into Image Pro 4.5 (Media Cybernetics Inc., Silver Spring, MD, USA) for quantitative analysis. Analytic variables included total area of new bone; total area of matrix; and total area of defect (defined by superimposed border region overlay). All images were calibrated for direct comparison. To determine the extent of bony bridging, data were examined as a function of new bone formed across the defect from one edge of the defect to the opposite. Briefly, each composite image was overlaid with a grid pattern consisting of 60 equally spaced columns oriented perpendicular to the surface of the dura. Bridging was expressed as the percentage of columns that contained new bone.

### Data analysis

All data were analyzed with GraphPad Prism v. 5.02 (GraphPad Software Inc., La Jolla, CA, USA). One-way analysis of variance (ANOVA) was performed using Tukey's method as the *post hoc* test for comparison of group means. Linear regression analysis was also performed on the ratio of new bone in the upper half of the scaffold to lower half as a function of therapy duration.
